# Combined Space and Alertness Related Therapy of Visual Hemineglect: Effect of Therapy Frequency

**DOI:** 10.3389/fnhum.2013.00373

**Published:** 2013-07-30

**Authors:** Walter Sturm, M. Thimm, F. Binkofski, H. Horoufchin, G. R. Fink, J. Küst, H. Karbe, K. Willmes

**Affiliations:** ^1^Department of Neurology, Clinical Neuropsychology, Section Neuropsychology, University Hospital RWTH University, Aachen, Germany; ^2^Department of Neurology, University Hospital Cologne, Cologne, Germany; ^3^Cognitive Neuroscience, Institute of Neuroscience and Medicine (INM3), Research Center Jülich, Jülich, Germany; ^4^Schmieder Clinic, Neurological Rehabilitation Centre, Allensbach, Germany; ^5^Neurological Rehabilitation Centre Godeshöhe, Bonn, Germany

**Keywords:** neglect, therapy frequency, therapy duration, alertness, optokinetic stimulation, spatial attention, reorganization

## Abstract

The combined efficacy of space- and alertness related training in chronic hemineglect was tested behaviorally and in a longitudinal fMRI study. Earlier results had shown that both space as well as alertness related training as single intervention methods lead to short term improvement which, however, is not stable for longer time periods. The neurobiological data obtained in these studies revealed differential cortical reorganization patterns for the two training approaches thereby leading to the hypothesis that a combination of both trainings might result in stronger and longer lasting effects. The results of our current study, however, – at least at first glance – do not clearly corroborate this hypothesis, because neither alertness training alone nor the combination with OKS on the group level led to significant behavioral improvement, although four of the six patients after alertness and even more after combined training showed a higher percentage of behavioral improvement than during baseline. Despite the lack of clearcut behavioral training induced improvement we found right parietal or fronto-parietal increase of activation in the imaging data immediately after combined training and at follow-up 3 weeks later. The study design had called for splitting up training time between the two training approaches in order to match total training time with our earlier single training studies. The results of our current study are discussed as a possible consequence of reduced training time and intensity of both training measures under the combined training situation.

## Introduction

A main symptom of hemineglect is a lack of exploration of space contralateral to the lesion. There are different theories for the explanation of hemineglect. Neglect symptoms can be seen as a deficit in processing and integration of contralesional sensory information (Kinsbourne, 1993; Fink et al., 2000). Some authors suggest an impairment of mental representation of space (Bisiach and Luzzatti, 1978; De Renzi, 1982). Karnath (1994a) hypothesized that damage to a neural egocentric reference system leads to neglect symptoms (transformation hypothesis).

Other theories emphasize deficits of spatial directing of attention to be correlated with the phenomenon of neglect (Posner et al., 1984; Kinsbourne, 1993). Following Heilman and Van Den Abell (1980) or Mesulam (1999) the left hemisphere controls spatial directing of attention only for the right half of space whereas the right hemisphere represents both sides. Thus, right hemisphere lesions have a stronger and more generalized impact on spatial attentional processing while deficits after left hemisphere lesions can be compensated for by the bilateral attention processing capacity of the right hemisphere.

Recent findings suggest that persisting neglect symptoms are not solely caused by dysfunction of specific cortical regions but rather by the disconnection of larger networks comprising partially distant frontal and parietal regions of the right hemisphere (Bartolomeo et al., 2007). A central role of the superior longitudinal fasciculus (SLF II) as a connection between these regions was demonstrated by stimulation of the SLF II during neurosurgical intervention in patients suffering from a temporal glioma (without neglect symptoms): stimulation led to a considerable rightward shift in a line bisection task (Thiebaut de Schotten et al., 2005).

These findings might be a direct anatomical counterpart to the hypothesis by Fernandez-Duque and Posner (1997) of a close cooperation between control systems for alerting and orienting, i.e., between anterior and posterior attention systems (see also Sturm et al., 2006a) and a disconnection of these systems could explain the strong correlation between non-spatial (vigilance or sustained attention) attention deficits and hemineglect after right hemisphere damage.

### Space-centered therapy approaches in hemineglect

Most clinical therapy methods for hemineglect aim at improving the patient’s exploration behavior. The following trainings led to amelioration of neglect symptoms although improvement was not stable over time: transcutaneous electroneutral stimulation of the left neck muscle (Karnath et al., 1993; Karnath, 1994b; Pizzamiglio et al., 1996); vestibular stimulation (Karnath, 1994b); visuomotor prism adaptation (Rossetti et al., 1998; Frassinetti et al., 2002, with repeated interventions yielding longer lasting effects); visual exploration training (Antonucci et al., 1995; Kerkhoff, 1998).

### Optokinetic stimulation therapy (OKS training)

Optokinetic stimulation is a procedure that displays visual stimuli on a screen which move coherently from the ipsilesional to the contralesional side thereby inducing smooth-pursuit eye movements if the patient follows the stimuli. This leads to an exogenously triggered directing of spatial attention to the neglected side.

Transient reduction of neglect under OKS has been demonstrated for the line bisection error (Mattingley et al., 1994), size, and space distortion (Kerkhoff et al., 1999; Kerkhoff, 2000), horizontal displacement of the sagittal midplane (Karnath, 1996), tactile extinction (Nico, 1999) as well as position sense deficit and motor weakness of the left limb (Vallar et al., 1993, 1995, 1997a,b). Unlike these studies, where OKS produced a passive, automatic stimulation via background movements, while patients were simultaneously engaged in another task, Kerkhoff et al. (2001, 2006) asked for active pursuit of the stimuli presented on the screen. After therapy, patients showed substantial improvement in digit cancelation, line bisection, visual size distortion, neglect dyslexia, and auditory neglect. These effects remained stable at a 2-week follow-up assessment. Compared to a conventional visual scanning training, OKS treatment showed stronger and more stable effects.

In our own therapy study (Sturm et al., 2006b; Thimm et al., 2009) seven neglect patients were treated daily for 45 min over a time period of 14 days with the OKS Training method introduced by Kerkhoff et al. (2001, 2006). After therapy, they showed a significantly higher number of improvements in a number of neglect tests (NETs) than after a 3 week baseline phase. Four weeks after the end of the training, however, lasting improvements could only be demonstrated in three of the patients. Longitudinal fMRT activation examinations revealed that a reduction of neglect symptoms after OKS training was accompanied by bilateral reactivation of parts of the posterior attention network (precuneus).

### Alertness related therapy approaches of spatial hemineglect

The presence and severity of spatial awareness deficits in hemineglect seem to depend greatly on the amount of attentional resources available for performance and thus can be strongly influenced by task demands (for a review see Bonato, 2012). Thus, spatial neglect subsequent to right hemisphere lesions often is closely associated with non-spatial deficits of attention like intrinsic alertness and sustained attention (Samuelson et al., 1988; Robertson, 1993, 2001; Hjaltason et al., 1996; Husain and Rorden, 2003; Corbetta and Shulman, 2011). Several studies have shown that the degree to which sustained attention is impaired is a strong predictor for the persistence of neglect (Samuelson et al., 1988; Robertson et al., 1997). The postulated interaction between an anterior alerting and a posterior spatial attention network (Heilman et al., 1978; Posner and Petersen, 1990; Fernandez-Duque and Posner, 1997; Sturm et al., 2006a) directly leads to the hypothesis that training of alertness may improve spatial neglect in right hemisphere stroke patients. First evidence supporting this hypothesis comes from a study by Robertson et al. (1995). In that study, attention training based on a self-instruction technique and on an enhancement of “phasic” alertness resulted in an improvement of neglect symptoms in all patients. Patients during the training were taught to give themselves the (silent, internal) instruction “be alert” before starting a task. In another study, Robertson et al. (1998) temporarily reduced the spatial bias of neglect patients by phasic alerting.

The concept of “alertness” on the one hand comprises a state of general wakefulness (tonic alertness) and the ability of top-down control of this state during phases of diminished external stimulation (Sturm et al., 1999, 2004b). On the other hand “phasic alertness” represents the ability to shortly improve the arousal level after a warning cue. In their rehabilitation study, Robertson et al. (1995) tried to activate the phasic alerting system, which may be intact, at least in part, after right hemisphere lesions (Sturm and Willmes, 2001; Yanaka et al., 2010) by using self-instructions. Degutis and Van Vleet (2010) found an improvement of sustained attention and neglect after a combined tonic and phasic alertness training (TAPAT).

In 1993 we (Sturm et al., 1993) developed a computerized training (AIXTENT) addressing different attention functions. During the AIXTENT alertness training, a car or motor cycle – driving at high speed – has to be stopped by the patient whenever an obstacle appears on the road. The impact of a 14-days treatment by this alertness training (45 min per day) on neglect initially was tested in a single case study (Sturm and Willmes, 2001) and later on in another study of seven neglect patients (Thimm et al., 2006). There was a significantly higher number of improvements after therapy than after a 3-week baseline phase, accompanied by significantly enhanced activations in the middle and medial frontal gyrus, in the anterior cingulate gyrus and in the right angular gyrus. The behavioral and functional changes, however – as for the OKS training (see above) – did not prove stable over a prolonged time period (3 weeks after the end of the therapy). There were, however, considerable interindividual differences, and in some patients (three out of seven) a stable effect of the alertness training on neglect symptoms in fact could be observed. Bilateral high frontal and anterior cingulate as well as left parietal reactivations corresponded to these long term effects and may represent a long lasting reorganization of the system for the top-down control of alertness.

### Comparison of alertness and OKS training effects

Behaviorally, OKS and Alertness training led to comparable functional improvements (Thimm et al., 2009). A comparison of the patterns of functional reorganization after the two training approaches revealed a frontal increase of activation after alertness training and a superior parietal increase of activation after OKS training, thus being consistent with the theory of interacting anterior intensity and posterior orienting attentional networks (Fernandez-Duque and Posner, 1997). From the results it became evident that both space as well as attention/alertness related training approaches as single interventions lead to a more or less comparable short term improvement of neglect symptoms but that neither of the two could induce lasting, i.e., long term effects. The data furthermore suggest that the differential activation of frontal or parietal areas may reflect the specific impact of the two types of training either on an anterior system for the control of attention intensity (AIXTENT) or on the posterior system of spatial attention (OKS), respectively. Thus, a combination of both therapy approaches might lead to a supplementary or even reinforcing effect. Indeed, other studies have shown that more permanent training effects in neglect patients can be achieved by the combination of different training methods. The combination of two space related trainings [visual exploration and limb activation training (Brunila et al., 2002) or neck muscle vibration (Schindler et al., 2002)] was particularly successful. A similar long lasting effect was seen after combined limb activation and sustained attention training (Wilson et al., 2000). Accordingly, the goal of our present study was to examine the efficiency of a combined alertness and OKS training in patients suffering from visual hemineglect.

## Materials and Methods

### Study design

**Figure 1:**
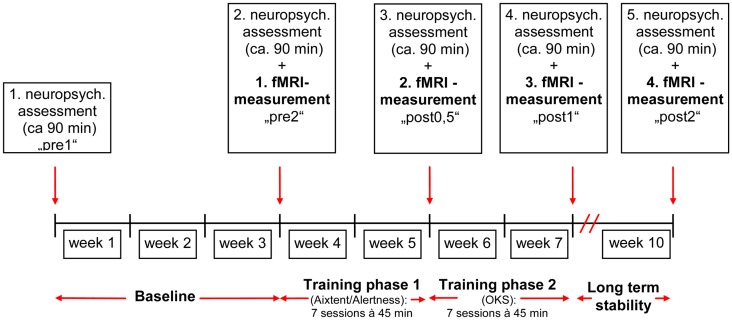
**Time schedule for the combined training alertness-OKS**.

The study design was comparable to our previous studies (Thimm et al., 2006, 2009) where we used either an alertness training as part of the computerized attention training system AIXTENT or an “OKS” training, but this time combining the two training methods. In order to keep the overall training time comparable to our former studies, the total training time was split between alertness and OKS training (see Figure 1). The study of patients started with a neuropsychological assessment of the neglect symptoms (“pre 1”). Neglect tests were repeated after 3 weeks in order to generate a baseline for the behavioral data (“pre 2”). This baseline served to control for behavioral improvements due to spontaneous recovery (although the fact that only patients in the postacute phase were included made spontaneous recovery effects less probable). When the inclusion criteria still held at the end of the baseline period, the first fMRI measurement took place, using a spatial attention paradigm (see below). During the following 4 weeks (excluding weekends and days reserved for Neuropsychological assessment or fMRI, see Figure 1), patients underwent seven sessions of alertness training followed by seven sessions of “OKS” training daily, each session lasting 45 min. We always started with the alertness training, because theoretically this is the more basic training procedure possibly enhancing overall activation level and thus enabling OKS training to be based on an improved level of arousal control. Immediately after the alertness training (“post 0.5”), at the end of the OKS period (“post 1”) and 3 weeks after the complete training period (“post 2”), again a neuropsychological and an fMRI assessment were carried out to assess both specific and combined short and long term effects of alertness and alertness + OKS training on spatial neglect.

### Patients

**Figure 2:**
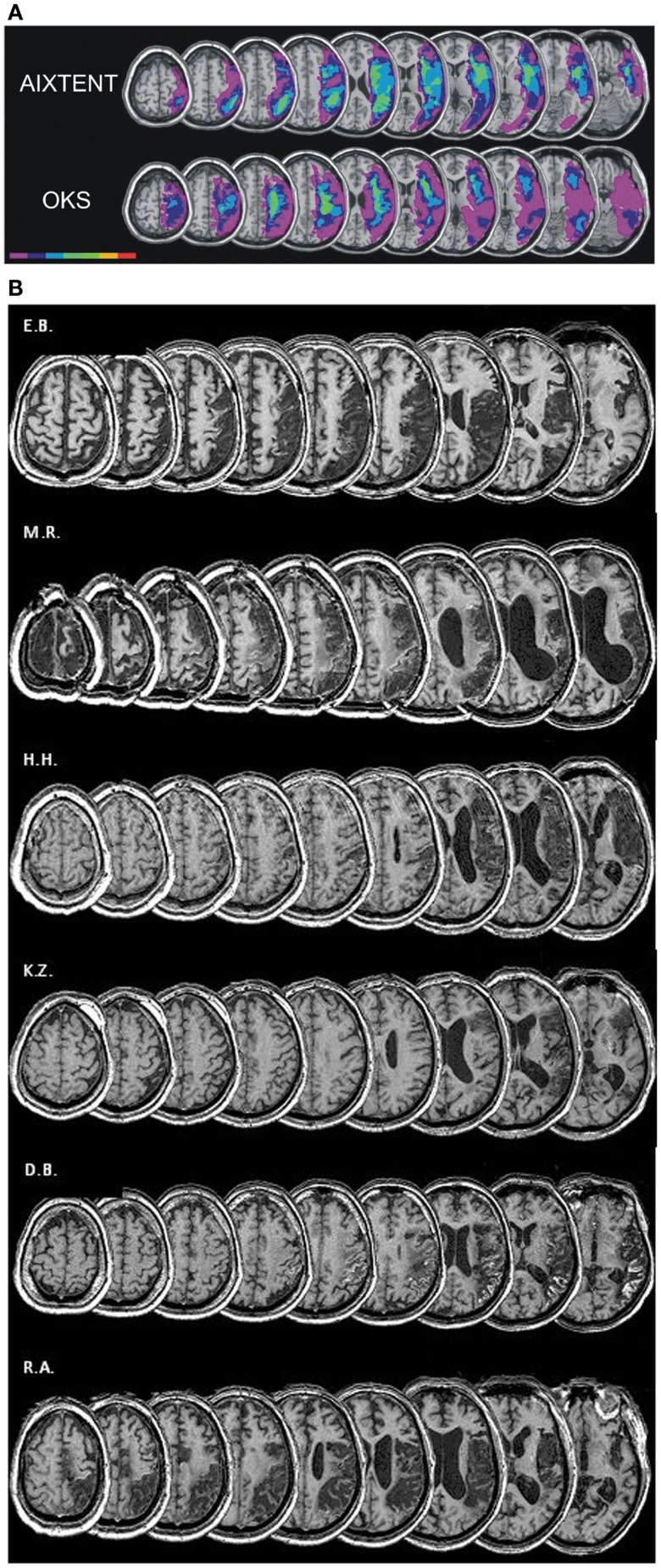
**(A)** Overlay lesion plots for the AIXTENT (alertness-training) group (*n* = 7) and OKS group (*n* = 7; Thimm et al., 2009). The number of overlapping lesions is coded and indicated by the color bar from violet (*n* = 1) to red (*n* = 7). **(B)** Lesion plots of individual patients of the combined alertness + OKS training group.

Six (two female, four male) right-handed patients [as assessed by a German translation of the Edinburgh-Handedness-Inventory (Oldfield, 1971)] with cortical and subcortical right hemisphere vascular lesions and symptoms of visuospatial neglect were included. The patient characteristics are detailed in Table 1. Median age was 62.5 years (range 45–74 years). All patients showed stable neglect symptoms for at least 3 months post stroke (median time 4 months, range 3–6 months). For inclusion, at the second pretest (“pre 2”) patients had to show neglect symptoms in at least two tasks of the “NET” (Fels and Geissner, 1996) or the “Test Battery of Attentional Performance” (TAP: Zimmermann and Fimm, 2007) described in detail later. Exclusion criteria were left-handedness, left hemisphere infarction, epilepsy, and any severe internal medical disease. Inclusion and exclusion criteria were the same as in our earlier studies (Thimm et al., 2006, 2009). Patients again were recruited from the inpatient service of the Neurological Clinic at the University Hospital Aachen and from the Neurological Rehabilitation Centre “Godeshöhe” in Bonn. The study was approved by the local Ethics Committee of the Medical Faculty of the University Hospital Aachen. Informed consent was given by all patients prior to participation in the study. Compared to our previous training studies (Thimm et al., 2006, 2009), the patients’ sample was similar with respect to sex distribution, age, and lesion localization. Figure 2B depicts the individual lesion plots. Each patient had a typical infarction of the right middle cerebral artery (MCA). The patients had frontoparietal (M.R, H.H.), fronto-temporo-parietal (E.B., K.Z.), or temporoparietal (D.B., R.A.) lesions. In four patients (E.B., H.H., D.B., R.A.) the lesions protruded into subcortical areas, probably comprising the SLF II, thus possibly causing a parieto-frontal disconnection. Interestingly, these four patients revealed the highest number of impaired test results in our neglect test battery (see Table 1). For comparison Figure 2A shows the lesion data of the patients included in our former two studies.

**Table 1:** **Patient characteristics and test results at the first pretest “pre 1”**.

Pat.	Sex	Age (years)	TPO (m)	NET LeC	NET LiC	NET SC	NET LB	NET Te	TAP VF (%)	TAP VF (RT)	TAP NEG (%)	TAP NEG (RT)	TAP VS
E.B.	F	45	6	+	−	−	−	−	+	−	+	−	−
M.R.	M	45	4	−	+	***−***	−	+	+	−	+	−	+
H.H.	M	74	3	***−***	+	+	+	−	+	***−***	+	−	−
K.Z.	M	69	3	***−***	+	+	+	+	+	−	+	−	***−***
D.B.	F	71	4	***−***	−	***−***	***−***	−	−	−	−	n.d.	−
R.A.	M	56	4,5	−	−	−	−	***−***	***−***	***−***	***−***	−	n.d.

*+, normal score; −, pathological score; **bold, significantly improved from “pre 1” to “pre 2;”** pat, patient; TPO, time post onset of neglect (months); NET, “Neglect-Test” (Fels and Geissner, 1996); LeC, letter cancelation; LiC, line cancelation; SC, star cancelation; LB, line bisection; Te, text; n.d., not done; TAP, “Test Battery of Attentional Performance” (Zimmermann and Fimm, 2007); TAP VF (%), visual field – % of detected left sided stimuli; TAP VF (RT), visual field – median reaction time (ms) on left sided stimuli; TAP NEG (%), neglect task – % of detected left sided stimuli; TAP NEG (RT), neglect task – median reaction time (ms) on left sided stimuli; TAP VS, visual scanning – overall number of detected stimuli in the left two columns*.

### Alertness training (CogniPlus)

The alertness training consisted of a subprogram of the Attention Training Program Package CogniPlus (Version 2.01: Sturm, 2007) and was developed from the AIXTENT alertness training described in the introduction. The patient watches on a computer screen a moving motorcycle from the driver’s viewpoint in a realistic scene. Sudden events such as falling trees or rocks, cars crossing the street, traffic lights changing to red and animals crossing have to be responded to as fast as possible by pressing a large response key. The task mainly follows the theoretical framework of an alertness task (simple reaction time measurement mostly without need for a selection of targets: targets are easily detectable and there is not much need for a discrimination between target and non-targets). A recent study has shown that both this alertness training and a classical alertness task (simple visual reaction time measurement without warning) activate very comparable cortical and subcortical networks (Clemens et al., 2013).

There are two different modes of the training: (a) Training of phasic alertness: in order to evoke phasic alerting, the participant hears a warning signal and sees a traffic sign announcing possible target situations before the actual event happens. Feedback is given visually if an obstacle is overlooked or if the response was too slow. This feedback ensures that participants know when they have made an error so that they can try to improve their performance. (b) Training of intrinsic alertness: under this training condition, no warning signals are given in order to provoke an improvement of intrinsic, i.e., top-down controlled alertness. Furthermore, under the intrinsic alertness condition the whole scene is made less clearly visible (foggy) in order to prevent phasic alerting signals to be evoked by the surroundings.

Under both conditions the difficulty level is adjusted by the average speed of the motorcycle. To reach a specific level, a minimum response time is necessary ranging from 1.8 s for the lowest to 0.3 s for the highest level. Depending on the subject’s mean response time the difficulty level is adapted automatically by the computer program. Before starting the training, during an instruction and practice period the mean response time of the patient is assessed which, in turn, defines the initial difficulty level for the subsequent training period.

### Optokinetic stimulation training

The OKS training used is part of the treatment program “EYEMOVE”^1^. Patients had to look at a computer screen (43° × 35°) where a pattern of randomly distributed, colored squares moving coherently from the right to the left side was displayed against a dark background. Patients were instructed to perform smooth-pursuit eye movements following the stimulus pattern until reaching the left margin and then to jump back to the right margin repeatedly. No head movements were allowed. To keep patients motivated, every few minutes the stimulus pattern was varied in color, speed (5–35°/s), size (0.2–2.5°), and number (30–70) of squares. The duration of each training session was 45 min. Every 10 min or whenever a patient asked for it, a break was allowed for a few minutes.

### Neuropsychological assessment

Neglect symptoms were assessed using subtests of the TAP [(Zimmermann and Fimm, 2007) subtests “neglect,” “visual field,” and “visual scanning”] and the NET (Fels and Geissner, 1996), a German version of the “Behavioral Inattention Test” (BIT: Wilson et al., 1987), including letter, star and line cancelation, line bisection, and text reading (see also Table 1).

The subtests of the TAP repeatedly have proven their sensitivity as control tasks in attention rehabilitation studies (e.g., Sturm et al., 1997, 2003; Sturm et al., 2004a).

#### TAP, subtest “neglect”

Patients were instructed to fixate on a central square (size 3.8°) on a black screen. To ensure steady fixation, they had to read aloud single letters appearing and changing every few seconds in the central square. Around the square in each visual hemifield the display showed 24 randomly distributed white distractors (small, hardly legible two, and three digit numbers). These stimuli were introduced to enhance possible neglect symptoms by distraction. In the gaps between these distractors a peripheral three digit target appeared at random locations in either the left or the right visual field within 13° from the central square. These three digit targets, however, appeared as flickering stimuli. Patients were instructed to press a key with the right index finger as soon as they detected the target. This was presented until the key was pressed or for a maximum of 3 s. In each visual half field 22 targets were presented at different positions. Dependent variable was the number of detected stimuli in the left visual half field.

#### TAP, subtest “visual field”

This test was very similar to the TAP-neglect test described above. In contrast to the neglect test, however, the screen was not filled with distractors. Thus stimuli could be detected more easily (as no distraction occurred). Forty-six stimuli were presented in each visual half field.

#### TAP, subtest “visual scanning”

Patients had to detect a target stimulus in a 5 × 5 matrix of similar distractors. The target stimulus was a square with an opening in the top line while the distractors had an opening in the left, right, or bottom line. Altogether 100 matrices were presented, half of them containing a target stimulus. Target stimuli were randomly distributed over the matrices, appearing two times at each possible position, thus 10 stimuli per column were presented. Patients were instructed to scan the matrix as fast as possible from the top left to the bottom right. They had to respond with their right hand by pressing either the left (“yes”) or the right (“no”) of two response keys deciding if the matrix contained a target stimulus or not. Dependent variable was the overall number of detected stimuli in the left two columns.

#### NET, cancelation tasks

These tasks required the patients to detect and cancel target stimuli distributed on a piece of paper. Dependent variable was the number of detected stimuli in the left half of the template.

*Letter cancelation:* targets: letter “E” or “R” (20 left, 20 right); with other letters serving as distractors.

*Line cancelation:* targets: lines of 26 mm length, rotated in different orientations (18 left, 18 right); no distractor.

*Star cancelation:* targets: little stars (28 left, 28 right); bigger stars, letters, and words served as distractors.

*NET, line bisection:* to assign the center of three lines of 20 cm length, located at the right, middle, and left side of an A4 sized sheet of paper. Dependant variable was the average deviation in millimeters across the three lines transformed into a percent score (100% = no deviation).

*NET, text reading:* to read aloud a newspaper article arranged in three columns.

### fMRI activation tasks

#### Spatial attention task

A modified version of the subtest “neglect” of the TAP was used as activation paradigm in a box-car fMRI design. The task stimuli were presented via a head mounted video optical unit (VisuaStim XGA with eye tracker, Arrington Research Inc.). Patients were instructed to fixate on a central square. In each visual hemifield, the display contained 24 randomly distributed white distractors (“#”). In the gaps between these distractors, a peripheral flickering target (as well “#”) appeared at random locations in either the left or the right visual field within 13° from the central square. The display covered a visual angle of 19.5° vertically and 30° horizontally. Each stimulus subtended 1.5° of visual angle. Target stimuli were presented in a pseudo-randomized sequence at varying positions in the left or right half of the screen. There were equal numbers of left- and right-sided targets (22 each). Stimulus onset asynchronies varied between 1500 and 4000 ms.

Patients were instructed to press a non-magnetic air pressure key with their right index finger as soon as they detected the target, which was presented until the key was pressed or for a maximum of 3 s.

#### Alertness task

This task was used to control for primary sensory and motor activation and for the alertness aspects of the neglect task (Sturm et al., 2006b). Patients had to respond to the same stimuli as in the neglect task. The only difference in the alertness task was the *location* of the stimuli, which were exclusively presented centrally, i.e., inside the fixation square. This condition undoubtedly also calls for some kind of spatial attention but this is much more focused centrally whereas under the neglect task condition a spatial distribution of attention is necessary (Sturm et al., 2006b).

### fMRI data acquisition

Each fMRI session consisted of two functional runs (alertness task, neglect task) in a box-car fMRI design which included 11 alternating periods of six times rest (15 s) and five times activation (37 s). Before each run, patients were informed about which kind of task would follow next. FMRI was performed on a 1.5 T Philips NT Gyroscan using a standard bird-cage head coil and T2^∗^-weighted gradient echo EPI sequences (TR: 2900 ms, FA: 90°, Matrix 64 × 64, FOV: 250mm × 250 mm, 31 continuous slices parallel to the AC-PC line, comprising the whole brain, slice thickness 3.5 mm, no inter-slice gap).

### Statistical analysis of the behavioral data

Test results were considered as indicative of neglect if they were below the test norm for healthy subjects (cancelation tasks, line bisection) or if the number of detected words/stimuli (text, TAP tests), or median reaction times (TAP visual field and NET) were significantly lower or slower, respectively, on the left than on the right side. This was assessed using Fisher’s exact test or by *t*-Tests.

For the individual patient, improvements in text reading, the cancelation tasks of the NET, the neglect specific subtests of the TAP, and the fMRI neglect task were investigated by Fisher’s exact test considering the number of left sided detected or canceled stimuli. Furthermore, cancelation tasks and line bisection (mean deviation from center to the right in millimeters transformed into a percent score: 100% = no deviation) were judged as improved, when a pathological score increased to within the normal range. Response times in the TAP and fMRI tasks were compared across the test sessions by means of ANOVA always considering only the left part of each test.

Due to this evaluation approach, patients served as their own control. As in our previous studies, the total number of improved test results after each training period was compared by Fisher’s exact test with the number of improvements at the end of the baseline period. Tests showing normal results from the beginning and thus allowing no neglect related improvement were not considered.

Additionally, the percentage of improved vs. not improved test results was compared across patients between the baseline and different training phases by means of Wilcoxon’s signed ranks test.

### Statistical analysis of the fMRI data

Analysis of the activation data was carried out using statistical parametric mapping software (SPM5, Wellcome Department of Imaging Neuroscience, London, UK^2^) using MATLAB version 6.5 (The MathWorks Inc., Natick, MA, USA). After discarding the first three volumes of each run, functional images were realigned to the new first scan of a session to compensate for movement artifacts. Realignment parameters showed no major translation (>one voxel size) or rotation (>2°), thus there was no reason to exclude any measurement. For the group analyses, realigned images were normalized to a standard EPI template based on the MNI reference brain following the Talairach convention (Talairach and Tournoux, 1988) resulting in a voxel size of 3mm × 3mm × 3 mm. To avoid image distortion caused by the lesions of the patients’ brains, only affine normalization was chosen. Finally, all images were smoothed with a Gaussian filter of 8 mm to improve signal-to-noise ratio.

In order to assess the neural correlates of behavioral short term improvements induced by each single training, group contrasts were set up between post 0.5 and pre 2 (AIXTENT) as well as post 1 and post 0.5 (OKS). The effect of the entire training program was investigated by the contrast post 1 vs. pre 2. Long term effects were investigated by the contrast post 2 vs. pre 2. Contrasts were controlled for deactivation by using an inclusive masking procedure. Only clusters comprising at least 10 voxels with a threshold of *p* < 0.05 false discovery rate (FDR)-corrected will be reported.

## Results

### Behavioral effects of alertness training, OKS training, and the combination of both

As pointed out above, our design enabled us to use each patient as his or her own control. Thus, we compared short term effects resulting from the CogniPlus alertness training (post 0.5 vs. pre 2), the combined effects of Alertness plus OKS training (post 1 vs. pre 2), and long term effects (post 2 vs. pre 2) with any spontaneous changes during the baseline period (pre 2 vs. pre 1). Significant improvement during the baseline was found in a total of 12 of 38 originally impaired neglect scores across the six patients (see Table 2). After the training periods, ameliorations were found in 12 of 39 test scores (CogniPlus alertness), and 17 of 39 test scores (CogniPlus alertness + OKS). Four weeks after the end of the last training procedure (long term effects) 13 out of 39 test scores remained improved. Tables 2 and 3 show the original results of the different neglect tasks for the different training periods and Table 4 presents the respective number of improvements resp. lack of performance changes plus the results of Fisher’s Exact Test. For comparison, Table 4 also presents the results of our former studies. In contrast to our former studies, neither alertness training alone nor the combination of alertness plus OKS training led to a significantly higher rate of improvement than the one caused by spontaneous remission in the baseline phase.

**Table 2:** **Results. of paper and pencil tasks**.

Pat.	CT letters					CT lines					CT stars					LB					Text				
	a	b	c	d	e	a	b	c	d	e	a	b	c	d	e	A	b	c	d	e	a	b	c	d	e
E.B.						83	n.d.	**100**	**100**	78	93	n.d.	89	**96**	**100**	67	n.d.	**100**	78	**100**	0	n.d.	**96**	**55**	**100**
M.R.	90	80	85	90	70											67	56	44	**89**	78					
H.H.																					100	55	55	**100**	**100**
K.Z.																									
D.B.	5	40	**80**	15	0	67	83	67	89	72	59	85	44	85	41	0	33	33	0	0	0	0	0	0	0
R.A.	70	35	50	**70**	**75**	72	78	67	94	89	81	70	63	81	74	67	67	89	56	89	0	55	55	55	55

***Bold, significant improvement compared to pre 2 (pre 1 in patient E.B.)**; empty cells, not impaired at pre 2 (pre 1 in patient E.B.); n.d., not done; CT/text, cancelation tasks and text: % of detected left sided stimuli/words; LB, line bisection: score = average deviation in millimeters across the three lines transformed into a percent score (100% = no deviation); a, pre 1; b, pre 2; c, post 0.5; d, post 1; e, post 2*.

**Table 3:** **Results of computerized tasks**.

Pat.	TAP VF (%)					TAP VF (RT)					TAP NEG (%)					TAP NEG (RT)					TAP VS				
	a	b	c	d	e	a	b	c	d	e	a	b	c	d	e	a	b	c	d	e	a	b	c	d	e
E.B.						785	n.d.	**448**	**553**	**512**						757	n.d.	930	755	712	50	n.d.	70	**100**	**95**
M.R.						558	506	**380**	**415**	**400**	91	82	77	**100**	95	704	1059	**519**	**586**	**486**					
H.H.						944	640	660	624	603	86	64	**86**	**91**	**91**	1103	1384	**1062**	**1157**	**817**	20	15	15	**45**	15
K.Z.						736	797	770	762	704						1180	966	968	**866**	**772**	45	70	75	**90**	75
D.B.	48	15	**41**	11	**48**	1378	1029	**725**	1770	**691**	5	0	0	0	5	n.d.	n.d.	n.d.	n.d.	n.d.	35	15	15	15	20
R.A.	78	93	96	89	91	962	672	693	621	696						1544	1459	**1052**	**773**	**968**	n.d.	20	15	15	30

***Bold: significant improvement compared to pre 2**; empty cells, not impaired at pre 2; n.d., not done; TAP, “Test Battery of Attentional Performance” (Zimmermann and Fimm, 2007); TAP VF (%), visual field – % of detected left sided stimuli; TAP VF (RT), visual field – median reaction time (ms) on left sided stimuli; TAP NEG (%), neglect task – % of detected left sided stimuli; TAP NEG (RT), neglect task – median reaction time (ms) on left sided stimuli; TAP VS, visual scanning – overall number of detected stimuli in the left two columns; a, pre 1; b, pre 2; c, post 0.5; d, post 1; e, post 2*.

**Table 4:** **Number of improved or unchanged test results after the different training periods (see Figure 1) and results of Fisher’s exact test for the current and for the preceding studies**.

Training	Therapy phase	Comparison	Initial number of test results indicative of neglect (baseline: pre 1, training: pre 2)	Number of significantly improved test results per phase	Number of not improved test results per phase	Fisher’s exact test for the comparison baseline/training resp. training/training (alertness/OKS)
Alertness (14 training sessions; Thimm et al., 2009) *n* = 7	Baseline	Pre 2–pre 1	32	3	29	*p* = 0.025
	Training	Post 1–pre 2	31	10	21	
OKS (14 training sessions;Thimm et al., 2009) *n* = 7	Baseline	Pre 2–pre 1	33	8	25	*p* = 0.017
	Training	Post 1–pre 2	30	16	14	
Alertness + OKS (7 training sessions each) *n* = 6	Baseline	Pre 2–pre 1	38	12	26	*p* = 1.000
	Alertness	Post 0.5–pre 2	39	12	27	
	Alertness + OKS	Post 1–pre 2	39	17	22	*p* = 0.349
	Alertness + OKS long term	Post 2–pre 2	39	13	26	*p* = 1.000

The percentage of improved vs. not improved test results across patients was 38.5% for the baseline, 36.5% for the alertness training, 64.8% for alertness + OKS training, and 36.5% for the long term phase 3 weeks after the end of both training procedures. The comparison of these improvement rates between the different phases by means of Wilcoxon’s signed ranks test (one-tailed) revealed *p* = 0.078 for the comparison alertness with alertness + OKS and of *p* = 0.094 for alertness + OKS with the long term phase. All other comparisons were far from significant. Thus, in this analysis there was a trend for a higher percentage of improvements after the administration of alertness + OKS training than after alertness training alone and for an improvement decline during the long term phase after the end of both training procedures. The patients with the highest number of initially impaired test parameters tended to profit least especially from the combined training approach whereas the opposite pattern occurred for the initially less impaired patients as can be seen from the individual percentage improvement scores (percentage of number of improved test scores with reference to the number of impaired scores at the end of the baseline phase) in Table 5. Four of the six patients (E.B., M.R., H.H., and K.Z.) numerically either after alertness or after combined training showed a higher percentage of behavioral improvement than during baseline. Because not every patient underwent each of the several test procedures it is difficult to compare the sensitivity of the different tests to detect behavioral changes during therapy in the single case. It seems, however, that with the computerized tasks a higher number of significant changes could be detected in the single case (TAP Visual field, response times for left sided stimuli: three improvements after Alertness training, two after OKS; TAP-Neglect, response times for left sided stimuli: three improvements after Alertness training, four after OKS; TAP Visual Scanning, no improvement after Alertness training but three improvements after OKS). This single case analysis shows the same trend for a higher efficacy of Alertness + OKS training compared with Alertness Training alone as the above reported group analysis. In contrast, most of the paper-and-pencil Tests could detect behavioral changes only in one patient.

**Table 5:** **Initial severity of impairment (number of neglect tasks outside normal range) and percentage of improvement (compared to the number of impaired parameters at the end of the baseline pre 2) during the different treatment phases for each individual patient**.

Pat.	Number impair. param. at pre 1	Number improv. param. at pre 2	Number not improv. param. at pre 2	% Improv. param. during basel	Number impair. param. at pre 2	Number improv. param. at post 0.5	Number not improv. param. at post 0.5	% Improv. param. during alertn. training	Number improv. param. at post 1	Number not improv. param. at post 1	% Improv. param. during alertn. + OKS training	Number still improv. param. at post 2	Number no longer improv. param. at post 2	% Still improv. param. at post 2
**R.A**.	9	4	5	**44.4**	9	1	8	**11.1**	2	7	**22.0**	1	8	**11.1**
**D.B**.	9	3	6	**33.3**	9	3	6	**33.3**	0	9	**0.0**	2	7	**22.0**
**E.B**.	7	0	7	**0.0**	8	4	4	**50.0**	5	3	**62.0**	5	3	**62.0**
**M.R**.	5	1	4	**20.0**	5	2	3	**40.0**	4	1	**80.0**	2	3	**40.0**
**H.H**.	4	2	2	**50.0**	5	2	3	**40.0**	4	1	**80.0**	2	3	**40.0**
**K.Z**.	4	2	2	**50.0**	3	0	3	**0.0**	2	1	**67.0**	1	2	**33.0**

Percentage of improvement during baseline refers to pre 1.

In the fMRI neglect task, one patient (M.R.) showed significant behavioral improvement after alertness training and two other ones (H.H. and D.B.) after OKS Training (see Table 6).

**Table 6:** **Behavioral results in the fMRI tasks**.

Pat.	fMRI spatial attention (%)				fMRI spatial attention (RT)			
	Pre 2	Post 0.5	Post 1	Post 2	Pre 2	Post 0.5	Post 1	Post 2
E.B.	39	23	9	16	945	1425	1249	1265
M.R.	27	**64**	18	11	1412	**764**	1026	606
H.H.	0	5	**9**	**11**	n.d.	1707	2135	1120
K.Z.	34	18	7	7	1957	1278	1685	2158
D.B.	11	18	14	**30**	2317	985	1105	1797
R.A.	7	2	5	7	1669	1328	1152	2553

***Bold, significant improvement compared to pre 2**; fMRI spatial attention (%): % of detected left sided stimuli; fMRI spatial attention (RT), median reaction time (ms) of stimuli detected on the left side*.

### fMRI data

After alertness training alone, concordant with the preponderance of absence of improvement at the behavioral level, no significant changes of neural activity were found (contrasts post 0.5 > pre 2). After combined training (alertness + OKS) a significant increase of activity (see Table 7) in the right superior parietal lobule (BA7) could be observed (post 1 > pre 2). Despite the fact that at follow-up (post 2 > pre 2) behaviorally some of the training induced improvements decreased, we not only still found the above mentioned increased right superior parietal activity (BA7) but also an additional increase in activity in the left inferior parietal lobule (PF) and in the dorsolateral prefrontal cortex (DLPF, BA9).

**Table 7:** **Macroanatomical structure, cytoarchitectonical area (Area_cyto_), cluster size in voxel, MNI coordinates (*x*, *y*, *z*), and maximum *T* value (*T*_max_) of the local maxima from the direct contrasts of post combined training against baseline (post 1 > pre 2) and long term effects (3 > 1)**.

Local maximum in macroanatomical structure	Area_cyto_	Cluster size (voxel)	MNI coordinates			*T*_max_
			*x*	*y*	*z*	
**POST 1 > PRE 2**						
R. superior parietal lobe	SPL_7P	18	18	−72	57	3.93
**POST 2 > PRE 2**						
L. inferior parietal cortex	IPC_PFcm	13	−57	−45	36	3.93
R. superior parietal lobe	SPL_7P	23	15	−69	63	4.17
R. prefrontal cortex	DLPF BA9	7	36	45	33	3.91

*The significance level was set to *p* < 0.05, FWE corrected for small volumes using the image masks of the SPM Anatomy toolbox v1.8 (Eickhoff et al., 2005). A cluster size of ≥10 contiguous voxels extended the threshold. L., left; R., right*.

## Discussion

From the results of our previous studies (Thimm et al., 2006, 2009) it became evident that both space as well as attention/alertness related training approaches as single interventions lead to a more or less comparable short term improvement of neglect symptoms, but that neither of the two can induce long term effects. A comparison of the patterns of functional reorganization after the two training approaches revealed a stronger frontal increase of activation after alertness training and a stronger superior parietal increase of activation after OKS training. The data thus suggest that differential activation of frontal or parietal areas may reflect the specific impact of the two types of training either on an anterior system for the control of attention *intensity* (AIXTENT) or on the posterior system of *spatial* attention (OKS), respectively. Thus, it was our hypothesis for the present study that a combination of both training approaches might lead to a supplementary or even reinforcing effect. Other studies in fact corroborated this hypothesis: the combination of two space related trainings [visual exploration and limb activation training (Brunila et al., 2002) or neck muscle vibration (Schindler et al., 2002)] as well as a combined limb activation and sustained attention training (Wilson et al., 2000) led to more long lasting effects than the single training methods.

Thus, the main aim of this study was to prospectively investigate in right hemisphere stroke patients suffering from chronic spatial neglect the behavioral and neural effects (by fMRI) of a combined alertness and OKS training. As in our previous studies (Thimm et al., 2006, 2009) in which the effects of alertness training or of OKS were investigated separately, we applied a study design in which each patient served as his/her own control by comparing the effects of the single (only alertness training) or combined (alertness + OKS) treatment with a baseline phase. Furthermore, the study design enabled us to test for long time effects 3 weeks after the end of the last training procedure.

In our former studies, each training procedure was administered on 14 consecutive days (except weekends) for 45 min each day. In order to keep the overall training time comparable to our former studies, in our present study the total training time was split between alertness and OKS training. Thus, each patient underwent seven sessions of alertness training followed by seven sessions of “OKS” training, each session lasting 45 min.

Interestingly, in our current study we could not replicate our former behavioral findings, nor could we find a clearcut beneficial effect of the combination of the former successful therapy approaches although in four of the six patients there was a trend favoring the combined approach. Patients E.B., M.R., H.H., and K.Z. numerically showed a higher percentage of behavioral improvement after alertness and especially after combined training than during baseline. This was mostly reflected in the results of the computerized neglect tasks which showed a somewhat higher sensitivity for training induced changes. This higher sensitivity in contrast to paper-and-pencil tests might be credited both to a higher attentional load evoked by these tasks (Bonato et al., 2010) and by providing scoring measures that are sensitive to specific deficits (Bonato and Deouell, 2013). The patients with the highest number of initially impaired test parameters (their lesions protruded into subcortical areas, probably comprising the SLF II, thus possibly causing a parieto-frontal disconnection) tended to profit least especially from the combined training approach whereas the opposite pattern occurred for the initially less impaired patients. In contrast to the single case findings the statistical analysis of the group results did not reveal an unequivocally significant behavioral improvement beyond effects during the baseline.

Neurobiologically, in the fMRI results there nevertheless were significant changes in activation patterns both immediately after the end of the combined training (though not after alertness training alone) and at the end of the 3-week follow-up period (right superior parietal resp. right superior and inferior parietal and right dorsolateral). This finding, too, might be interpreted as a specific benefit of combined Alertness + OKS training.

Our three efficacy studies were quite comparable with respect to the initial severity of neglect symptoms or lesion characteristics: in all our studies, neglect patients presented with 32–38 test parameters indicative of neglect at the end of the baseline phase, all patients had typical infarctions of the right MCA. In our recent study there was, however, a tendency for patients showing a higher number of initially impaired neglect test scores to benefit least, especially from the combined training approach. This should be reconsidered in future studies with a higher number of patients showing a comparable initial level of impairment.

Studies on the efficacy of aphasia therapy revealed a clear cut correlation between intensity and duration of therapy and its efficiency (e.g., Bhogal et al., 2003; Neininger et al., 2004). Moreover, in a recent study dealing with the impact of attention therapy on language function in aphasic patients, the authors neither found improvement of attention nor of language functions (Graf et al., 2011), although the same attention training procedure had been shown to be efficient in a couple of studies before (e.g., Sturm et al., 1997, 2003; Plohmann et al., 1998). The authors discuss the lack of efficiency in their study in the light of training frequency leaving the patients with only half of the training time for each approach as compared to former efficacy studies. This situation is quite comparable to our training study where we split total training time between Alertness and OKS training with the consequence of a lack of clearcut functional improvement by the single and only a trend for higher efficacy of the combined training approaches. Thus, the critical parameter of therapy outcome might be total time spent for the training. This hypothesis is corroborated by the observation that in our recent study the highest percentage of behavioral improvement and significant functional reorganization was achieved at the end of the OKS training, i.e., at the point in time during our study, when the total training time (summed up for alertness + OKS training) reached the same amount as that for the individual training procedures in our former studies (Sturm et al., 2004a; Thimm et al., 2006, 2009). The results of our combined approach, however, do not allow the conclusion that it is the combination of alertness plus OKS training which might be more efficient than alertness training alone. It might be either the addition of the OKS treatment which increases efficacy or just the fact that alertness plus OKS treatment sum up for a more adequate overall amount of therapy. Our former studies revealed significant functional improvement for both therapy approaches after 14 training sessions each. Even summing up the efficacy of both training approaches in combination in our current study does not lead to a comparable behavioral effect as for each approach *per se* in the earlier studies. This observation, again, points to overall training time for each training procedure as the critical parameter. On the other hand, the fact that after the follow-up period (3 weeks after the end of alertness + OKS training) there was a right fronto-parietal reorganization pattern (thus combining the frontal reorganization after alertness plus parietal reorganization after OKS, see Thimm et al., 2009) might, however, mirror a combined training and not only a summed up training time effect. Anyway it might be desirable to do another study administering both training procedures in the opposite order starting with OKS training or combining both methods in every therapy session keeping overall therapy time constant. Our earlier studies have shown that specific training approaches – if administered for at least 14 consecutive training sessions – besides behavioral improvement lead to reactivation of parts of the originally involved functional brain networks. It seems that only prolonged intensive training of the impaired cognitive function can provoke cerebral reorganization procedures in the networks subserving the impaired function which also holds true in our current study. Earlier, this has been revealed in animal studies where intensive and long lasting stimulation led to an enlargement of cortical sensory and motor areas (Jenkins et al., 1990; Nudo et al., 1996) and in human subjects after somatosensory discrimination training (Braun et al., 1999). Thus, our results are relevant for the ongoing discussion about the link between intensity and duration of cognitive retraining procedures and outcome in cognitive rehabilitation.

## Conflict of Interest Statement

The authors declare that the research was conducted in the absence of any commercial or financial relationships that could be construed as a potential conflict of interest.

## Footnotes

^1^http://www.medicalcomputing.de

^2^http://www.fil.ion.ucl.ac.uk/spm
